# Memory-type ST2^+^CD4^+^ T cells participate in the steroid-resistant pathology of eosinophilic pneumonia

**DOI:** 10.1038/s41598-017-06962-x

**Published:** 2017-07-28

**Authors:** Naoko Mato, Kiyoshi Hirahara, Tomomi Ichikawa, Jin Kumagai, Masayuki Nakayama, Hideaki Yamasawa, Masashi Bando, Koichi Hagiwara, Yukihiko Sugiyama, Toshinori Nakayama

**Affiliations:** 1 Division of Pulmonary Medicine, Department of Internal Medicine, Jichi Medical University, 3311-1 Yakushiji, Shimotsuke-city, Tochigi 329-0434 Japan; 2 Department of Immunology, Graduate School of Medicine, Chiba University, 1-8-1 Inohana, Chuo-ku, Chiba, 260-8670 Japan; 3 Department of Respiratory Medicine, Nerima-Hikarigaoka Hospital, Tokyo, Japan

## Abstract

The lung develops an unique epithelial barrier system to protect host from continuous invasion of various harmful particles. Interleukin (IL-)33 released from epithelial cells in the lung drives the type 2 immune response by activating ST2− expressed immune cells in various allergic diseases. However, the involvement of memory-type ST2^+^CD4^+^ T cells in such lung inflammation remains unclear. Here we demonstrated that intratracheal administration of IL-33 resulted in the substantial increase of numbers of tissue-resident memory-type ST2^+^CD4^+^ T cells in the lung. Following enhanced production of IL-5 and IL-13, eosinophilic lung inflammation sequentially developed. IL-33-mediated eosinophilic lung inflammation was not fully developed in T cell-deficient *Foxn1* ^*nu*^ mice and NSG mice. Dexamethasone treatment showed limited effects on both the cell number and function of memory-type ST2^+^CD4^+^ T cells. Thus our study provides novel insight into the pathogenesis of eosinophilic lung disease, showing that memory-type ST2^+^CD4^+^ T cells are involved in IL-33-induced eosinophilic inflammation and elicited steroid-resistance.

## Introduction

Memory CD4^+^ T cells play a crucial role in the pathogenesis of chronic inflammatory lung diseases, such as asthma^1, 2^. Interleukin (IL-)33 is a member of the IL-1 family of cytokines and is a ligand for the ST2 receptor^3^. Extracellular IL-33 induces type-2 immune responses by activation of ST2 (the receptor for IL-33) expressed immune cells accompanied by a massive infiltration of eosinophils in mucosal sites^3, 4^. IL-33 activates the ST2-positive memory Th2 cell subpopulation to produce dramatically increased levels of IL-5^2, 5^. This indicates that the ST2-positive memory Th2 cell subpopulation is critical for the pathology of allergic inflammation and function as “memory-type pathogenic Th2 (Tpath2) cells”^2, 5, 6^. However, the mechanism by which memory-type ST2^+^CD4^+^ T cells present under normal steady-state conditions in the lung respond to IL-33 to induce eosinophilic inflammation *in vivo* remains unknown.

Emerging studies have revealed the pathogenic roles of IL-33 in allergic diseases. Genome-wide association studies have identified the *IL33* and *ST2* genes as major susceptibility gene loci in allergic diseases^7^. Eosinophilic pneumonia, which is induced by various airborne irritants, often requires high doses of steroids for the treatment of severe respiratory failure^8, 9^. However, eosinophilic inflammation frequently relapses when the steroid dose is tapered^8^. High levels of IL-33 and massive eosinophil infiltration in the bronchoalveolar lavage (BAL) fluid in patients with eosinophilic pneumonia suggest that the IL-33-ST2 axis is involved in the pathophysiology of eosinophilic pneumonia^10^. However, the cellular mechanisms underlying the IL-33-mediated pathology of eosinophilic lung inflammation have not been well elucidated.

In the present study, we examined pathogenic roles of memory-type ST2^+^CD4^+^ T cells in the IL-33-induced eosinophilic lung inflammation. Intra-tracheal administration of IL-33 resulted in increased numbers of lung tissue-localized ST2^+^CD4^+^ T cells with enhanced production of IL-5 and IL-13. In this IL-33-induced lung inflammation model, T cells rather than ILC2s are the major contributors in the pathology of eosinophilic inflammation. Interestingly, CD44^+^ST2^+^CD4^+^ T cells appeared to be resistant to the treatment of high dose dexamethasone. Thus, lung-resident memory-type ST2^+^CD4^+^ T cells could be a potential therapeutic target for the patients with steroid-resistant allergic inflammation such as eosinophilic pneumonia.

## Results

### IL-33 induced an increase in lung tissue-localized memory-type ST2^+^CD4^+^ T cells along with enhanced production of IL-5 and IL-13

**Figure 1:**
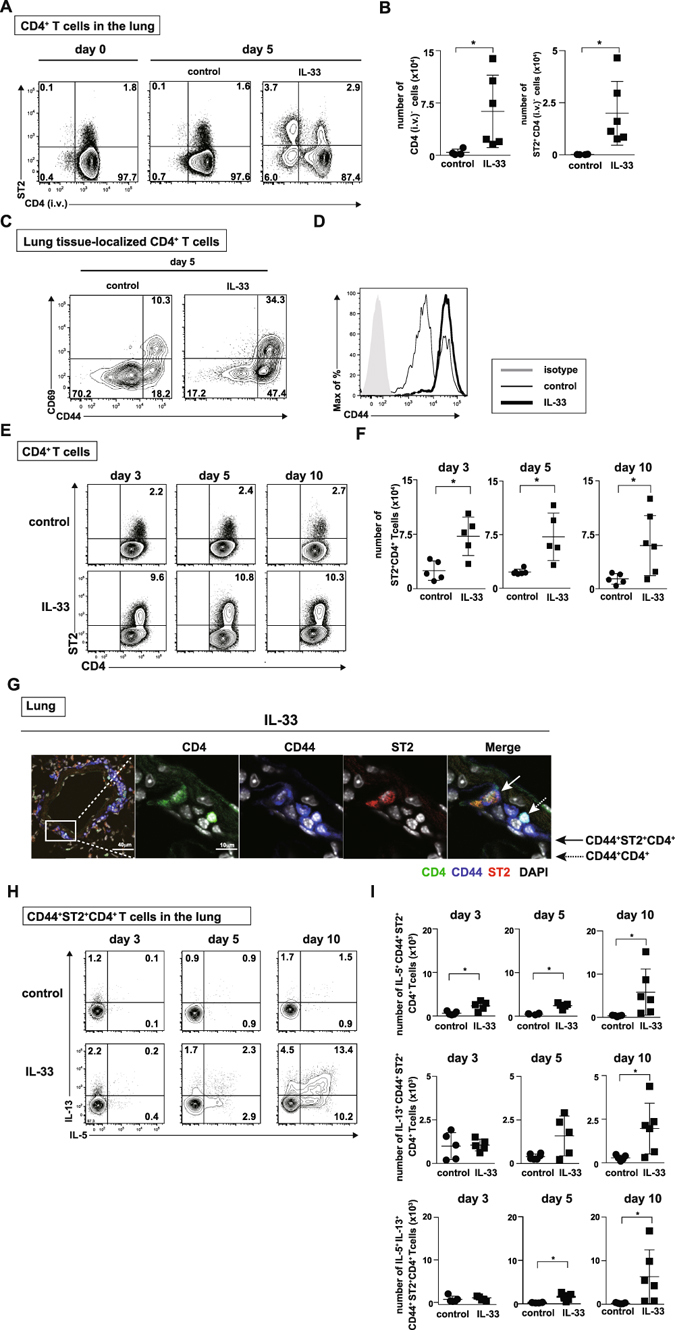
IL-33 induced increased expression of ST2 in lung tissue-localized memory-type ST2^+^CD4^+^ T cells along with enhanced production of IL-5 and IL-13. IL-33 or saline (control) was administered intratracheally to BALB/c mice on Day 0, and the indicated assays were performed on Day 3 to 10. (**A**) To distinguish lung tissue-localized CD4^+^ T cells and blood-borne CD4^+^ T cells, anti-CD4 antibody was injected intravenously and analyzed three minutes later. In the CD4^+^ T cells in the lung, injected antibody-unstained cells (CD4 (i.v.)^−^ cells) indicate lung tissue-localized CD4^+^ T cells. Representative flow cytometry plots of ST2^+^CD4 (i.v.)^−^ CD4^+^ T cells in the lung at the indicated time points. (**B**) Absolute numbers of CD4 (i.v.)^−^CD4^+^ T cells (left panel) and ST2^+^CD4 (i.v.)^−^ CD4^+^ T cells (right panel) in the lung from the two groups at day 5 are depicted. (**C**) Representative flow cytometry plots of lung tissue-localized CD4^+^ T cells in the lung at day5 after saline (control) or IL-33-admnistration are depicted. (**D**) The histogram represents the expression of CD44 on lung tissue-localized CD4^+^ T cells. (**E**) Representative flow cytometry plots of ST2^+^CD4^+^ T cells in the lung at the indicated time points. (**F**) Absolute numbers of ST2^+^CD4^+^ T cells in the lung from two groups at the indicated time points are depicted. (**G**) Representative confocal microscopic pictures of lung tissue stained with anti-CD4 (green), anti-CD44 (blue), and anti-ST2 (red) are shown. The solid arrow indicates CD44^+^ST2^+^ CD4^+^ T cells, and the dotted arrow indicates CD44^+^ CD4^+^ T cells. Scale bars, 40 μm and 10 μm. (**H**) Representative intracellular staining profiles of IL-5 and IL-13 are shown on CD44^+^ST2^+^ CD4^+^ T cells. (**I**) Absolute numbers of the indicated cytokine producing-CD44^+^ST2^+^ CD4^+^ T cells in the lung from the two groups at the indicated time points are depicted. The mean values ± SD are shown for five to six mice in each group. Three independent experiments were performed with similar results (**A–I)**. control: saline-treated mice, IL-33: IL-33-treated mice, *p < 0.05; Mann-Whitney U test.

IL-33 coordinates type 2 immune response and tissue repair in the mucosal barrier sites *in vivo* through the activation of ST2-positive immune cells^11^. To explore the non-redundant roles of IL-33 in CD4^+^ T cells in the mucosal barrier in the lung, we first assessed the expression of ST2 on CD4^+^ T cells in normal BALB/c mice under steady state conditions. We found higher percentages of ST2^+^CD4^+^ T cells in the lung than in the spleen (Fig. S1A and B). ST2^+^CD4^+^ T cells showed higher expression of CD44 and lower expression of CD62L than ST2^−^CD4^+^ T cells in the lung (Fig. S1C and D). Because the dynamics of IL-33-stimulated ST2^+^CD4^+^ T cells in the lung are unclear, we next examined the changes in the location and function of ST2^+^CD4^+^ T cells in the lung after intratracheal administration of IL-33. BALB/c mice were intravenously injected with anti-CD4 antibody and sacrificed three minutes later to distinguish between lung tissue-localized CD4^+^ T cells and blood-borne CD4^+^ T cells^12^. The majority of intravenously injected antibody-unstained cells were reported to be tissue-resident memory T cells^12, 13^. Most of CD4^+^ T cells in the lung mononuclear cell preparation on Day0 were in the lung vasculature and not in the tissue, because they were stained with anti-CD4 antibody given intravenously 3 minutes before sacrifice (Fig. 1A left). In contrast, five days after intratracheal administration of IL-33, substantial numbers of CD4^+^ T cells (Fig. 1A right panel and 1B) were found within the lung tissue. There were small changes in the phenotype of CD4^+^ T cells in the spleen or peripheral blood by the administration of IL-33 (Fig. S1E). IL-33 administration resulted in increased CD44^+^ and CD69^+^ cells among lung tissue-localized ST2^+^CD4^+^ T cells (Fig. 1C and D). Next, we performed experiments addressing the time course of ST2^+^CD4^+^ T cells in the lung after intratracheal administration of IL-33 (Fig. S1F). The number of ST2^+^CD4^+^ T cells in the lung was significantly increased at Day 3, and the accumulation of ST2^+^CD4^+^ T cells persisted for at least 10 days after intratracheal administration of IL-33 (Fig. 1E and F) (*P* < 0.05; Mann-Whitney U test). An immunohistological analysis of the lungs of mice that had been intratracheally administered IL-33 revealed the accumulation of CD44^+^ST2^+^ CD4^+^ T cells nearby bronchioles and blood vessels accompanied by the formation of lymphoid clusters (Fig. 1G). With regard to the function of ST2^+^CD4^+^ T cells, IL-33 stimulation generated limited numbers of IL-5 and IL-13 producers among CD44^+^ST2^+^ CD4^+^ T cells on Days 3 and 5 (Fig. 1H and I, left and middle panels). However, over the course of 10 days, the number of IL-5 and IL-13 producers among CD44^+^ST2^+^ CD4^+^ T cells was significantly increased (Fig. 1H and I, right panels) (*P* < 0.05; Mann-Whitney U test). IL-4 production from CD44^+^ST2^+^ CD4^+^ T cells was not altered by the administration of IL-33 (Fig. S1G).

It has also been reported that innate lymphoid type 2 cells (ILC2s), which express ST2, are another crucial target cells for IL-33 in mucosal barrier sites in the lung^14^. Consistent with the results of a previous report^15^, approximately 50% of ILC2s in the lung expressed ST2 without IL-33 stimulation *in vivo* (Fig. S1H and I (day 0)). IL-33 stimulation significantly increased the number of ST2^+^ILC2s on Day 3 (Fig. S1I and J). Consistent with this result, increased numbers of IL-5- and IL-13-producing ILC2s were detected even on Day 3 (Fig. S1K and L). Significantly increased numbers of cytokine-producing ILC2s were continuously detected on Day 10 (Fig. S1K and L) (*P* < 0.05; unpaired *t* test). Thus, IL-33 stimulation *in vivo* appeared to induce memory-type ST2^+^CD4^+^ T cells and ILC2s to generate IL-5 and IL-13 producers, however the time course of responses differed between memory-type ST2^+^ CD4^+^ T cells and ST2^+^ ILC2s.

### Intratracheal administration of IL-33 induced substantial infiltration of eosinophils in the lung

**Figure 2:**
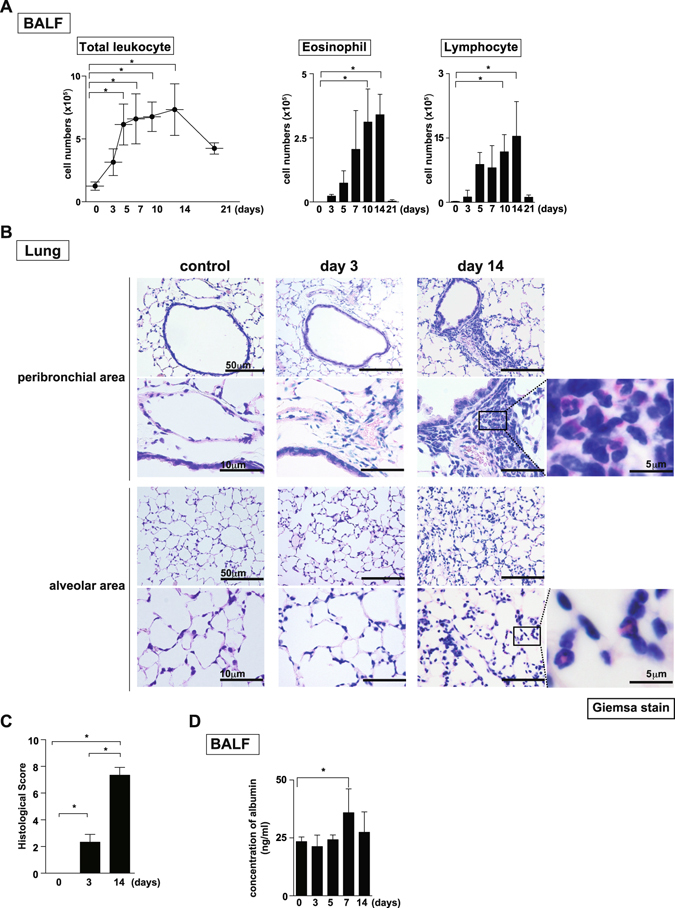
Intratracheal administration of IL-33 induced infiltration of eosinophils in the lung. IL-33 or saline (control) was administered intratracheally to BALB/c mice on Day 0, and the indicated assays were performed on Days 3 to 14. (**A**) The BAL fluid was assessed at the indicated time point. The numbers of total leukocytes (left), eosinophils (middle), and lymphocytes (right) in the BAL fluid are shown. (**B**) Microscopic pictures of the lungs from mice as in **A**, fixed and stained with Giemsa reagent. Scale bars: 50 μm (top and 3^rd^ line), 10 μm (2^nd^ and bottom line) and 5 μm (right panel of 2^nd^ and bottom line). (**C**) Histological scores from Giemsa-stained samples are depicted. (**D)** The vascular permeability was determined by the concentration of albumin in BAL fluid. Representative data were obtained from four independent experiments. Mean values ± SD are shown for (**A)** and (**D)**. *p < 0.05: one-way ANOVA.

We next examined the sequential pathophysiological changes in the lungs of mice that had received intratracheal administration of IL-33 (Fig. S2A). A significantly increased number of total inflammatory leukocytes in BAL fluid was detected from Day 5 to Day 14 (Fig. 2A left panel) (*P* < 0.05: one-way analysis of variance [ANOVA]). Absolute numbers of eosinophils and lymphocytes in the BAL fluid were significantly increased on Day 10 and 14 (Fig. 2A middle and right panels) (*P* < 0.05: one-way ANOVA). While, the number of total leukocytes, eosinophils, and lymphocytes in the peripheral blood remained almost the same after IL-33-administration (Fig. S2B). A histological analysis of the lungs of mice intratracheally administered IL-33 revealed few eosinophils and lymphocytes in the peribronchial and perivascular areas on Day 3 (Fig. 2B middle panels). Significant infiltration of eosinophils in the peribronchial area and alveolar spaces was confirmed on Day 14 in Giemsa-stained samples (Fig. 2B right column, 2 C) (*P* < 0.05: one-way ANOVA). The vascular permeability was significantly increased on Day 7 (Fig. 2D) (P < 0.05: one-way ANOVA). Altogether, mice intratracheally administered IL-33 showed massive infiltration of eosinophils in both the peribronchial space and lung parenchyma with enhanced vascular permeability without a systemic increase in numbers of eosinophils.

### IL-33-induced lung inflammation was severely attenuated in Foxn1^nu^ mice

Next, we wanted to determine which inflammatory cells were involved in the IL-33-induced eosinophilic inflammation using mice that genetically lack eosinophils (*Gata1* ^*tm6Sho*^ mice)^16^ or mast cells (*Kit* ^*W*^ */Kit* ^*W-v*^ mice)^17^. As expected, *Gata1* ^*tm6Sho*^ mice showed no increase in the number of eosinophils in the BAL fluid but a small number of infiltrated leukocytes after intratracheal administration of IL-33 (Fig. S3A, left panels, B and C). Mast cells are known to express ST2 and are able to respond to IL-33 stimulation^3^. Interestingly, however, *Kit* ^*W*^ */Kit* ^*W-v*^ mice showed similar increases in the number of leukocytes in the BAL fluid, especially eosinophils, to wild-type mice (Fig. S3A, right panels). Similar to the findings in the BAL fluid, we found substantial infiltrated leukocytes in both the peribronchial and alveolar areas of the lung of *Kit* ^*W*^ */Kit* ^*W-v*^ mice (Fig. S3D and E). These results indicate that eosinophils but not mast cells play an important role in the eosinophilic inflammation in the IL-33-induced lung inflammation model.

**Figure 3:**
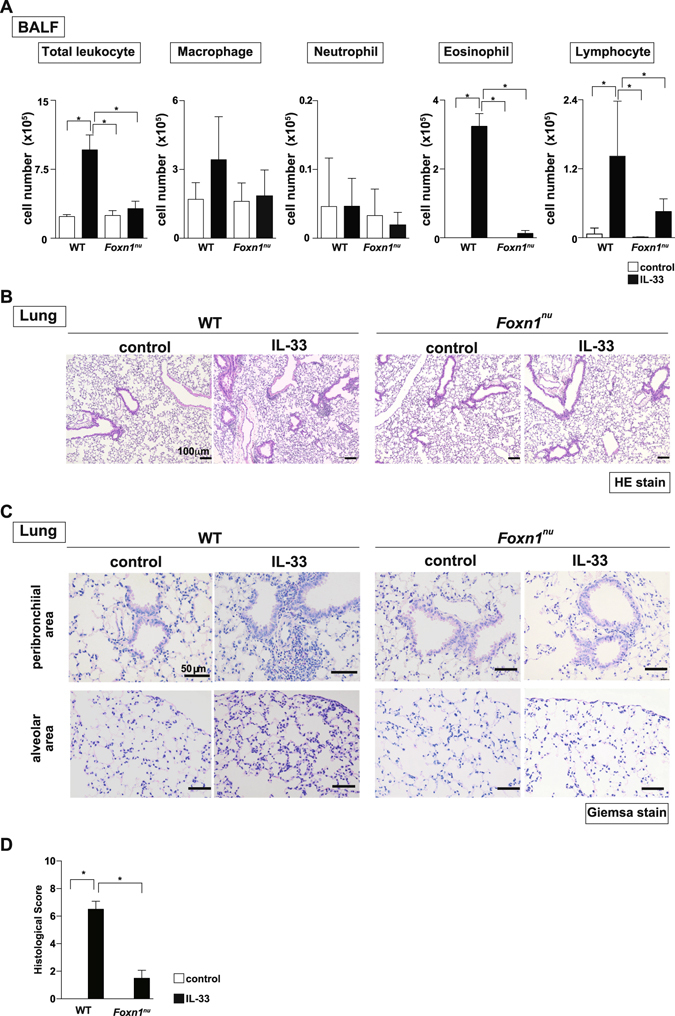
*Foxn1* ^*nu*^ mice showed attenuated eosinophilic inflammation induced by the administration of IL-33. IL-33 or saline (control) was administered intratracheally to BALB/c mice (wild type) or *Foxn1* ^*nu*^ mice on Day 0, and the indicated assays were performed on Day 14. (**A**) Absolute cell numbers of total leukocytes, macrophages, neutrophils, eosinophils, and lymphocytes in the BAL fluid are shown. The mean values ± SD are shown for four mice per group. (**B,C)** Representative histology of the lungs from mice as in **A**, fixed and stained with hematoxylin and eosin reagent (**B)** or Giemsa reagent (**C)**. Scale bars: 100 μm in (**B)**, 50 μm in (**C)**. (**D**) Histological scores from Giemsa-stained samples are depicted. Three independent experiments were performed, and similar results were obtained. *p < 0.05: one-way ANOVA.

We next examined whether or not CD4^+^ T cells are crucial for the pathology of eosinophilic lung inflammation induced by intratracheal administration of IL-33. The number of total leukocytes, eosinophils, and lymphocytes in BAL fluid was significantly decreased at Day14 in *Foxn1* ^*nu*^ mice, which are deficient in T cells^18^, compared to wild-type mice (Fig. 3A) (P < 0.05; one-way ANOVA). Similarly, the infiltration of inflammatory cells including eosinophils was apparently decreased in the peribronchial and alveolar areas of the lung of *Foxn1* ^*nu*^ mice compared to wild type mice (Fig. 3B,C and D). Next, we analyzed ILC2s in both wild-type and *Foxn1* ^*nu*^ mice. The number of ST2^+^ ILC2s induced by intratracheal administration of IL-33 was increased substantially in *Foxn1* ^*nu*^ mice compared to that in wild-type mice (Fig. S3F and G). While, the increase in the number of IL-5- and IL-13-producing ILC2s after IL-33 stimulation was almost equivalent between wild-type mice and *Foxn1* ^*nu*^ mice (Fig. S3H). These results indicate that the reaction of ILC2s to IL-33 was similar between wild-type and *Foxn1* ^*nu*^ mice at the late time point in our experimental model. Taken together, these findings suggest that the severity of IL-33-induced eosinophilic lung inflammation depends predominantly on T cells and not ILC2s at least at the peak of inflammation.

### CD4^+^ T cells but not ILC2s contribute to the pathogenicity of IL-33-induced lung inflammation

**Figure 4:**
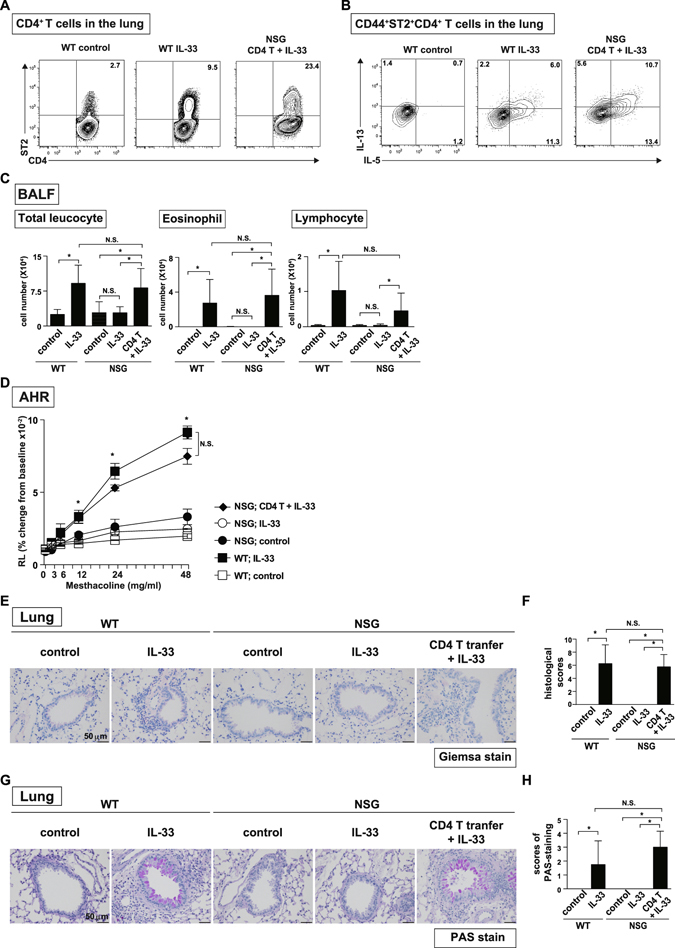
Adoptive transfer of CD4^+^ T cells restored IL-33-induced eosinophilic inflammation in NSG mice. (**A**) Representative flow cytometry plots of ST2^+^CD4^+^ T cells in the lung are shown. (**B**) Representative intracellular staining profiles of IL-5 and IL-13 are shown on CD44^+^ST2^+^ CD4^+^ T cells from wild type mice (BALB/c) and NSG mice with adoptive transfer. (**C)** Saline (control) or IL-33 was administered intratracheally to wild type (BALB/c) mice and NSG mice with adoptive transfer of CD4^+^ T cells and BAL was performed at day 14. The numbers of total leukocytes, eosinophils and lymphocytes in the BAL fluid are shown. *p < 0.05: one-way ANOVA. N.S. means not significant. (**D)** Lung resistance (RL) was assessed in response to the increasing doses of methacholine. The mean values (four mice per group) are shown with SDs. *p < 0.05: one-way ANOVA. N.S. means not significant. (**E**–**H)** Microscopic pictures of the lungs from mice, fixed and stained with H.E. (**E**) and PAS (**G**) reagent. Scale bars: 50 μm. Histological scores from HE-stained samples (**F**) and PAS-stained samples (**H**) are depicted.

To address the contribution of ILC2s to the pathogenicity of lung inflammation in our experimental setting, we examined the function of CD4^+^ T cells stimulated by IL-33 under ILC2-sufficient or ILC2-deficient conditions using mice genetically lacking ILCs, T cells, B cells, and NK cells (NSG mice) (Fig. S4A)^19^. Even in the absence of ILC2s, the numbers of ST2^+^CD4^+^ T cells were substantially increased in the lung after IL-33 stimulation in CD4^+^ T cell-transferred mice (Figs 4A, S4B and C). Lung tissue-localized CD4^+^ T cells in NSG mice showed a higher expression of CD69 and CD44 than in control wild type mice (Fig. S4D). CD44^+^ST2^+^CD4^+^ T cells in the lung showed a similar expression of Th2 cytokines, such as IL-5 and IL-13, to control wild type mice (Fig. 4B). NSG mice with adoptive transfer of CD4^+^ T cells showed similar numbers of infiltrated inflammatory cells in the BAL fluid to control wild type mice (Fig. 4C). Consistent with these results, the level of AHR in the NSG mice with transferred CD4^+^ T cells was similar to that in the control wild type mice (Fig. 4D). In contrast, NSG mice without adoptive transfer of CD4^+^ T cells showed a significant decrease in the number of infiltrated eosinophils in the BAL fluid and a decreased level of AHR after the intra-tracheal administration of IL-33 (Fig. 4C and D). Comparable levels of infiltration of eosinophils in the peribronchial area together with mucus hyperproduction were detected between NSG mice with adoptive transfer of CD4^+^ T cells and control wild type mice after IL-33 stimulation (Fig. 4E–H). These results indicated that CD4^+^ T cells but not ILC2s were essential for the induction of eosinophilic inflammation in our experimental setting.

### Lung-localized memory-type ST2^+^CD4^+^ T cells were steroid-resistant

**Figure 5:**
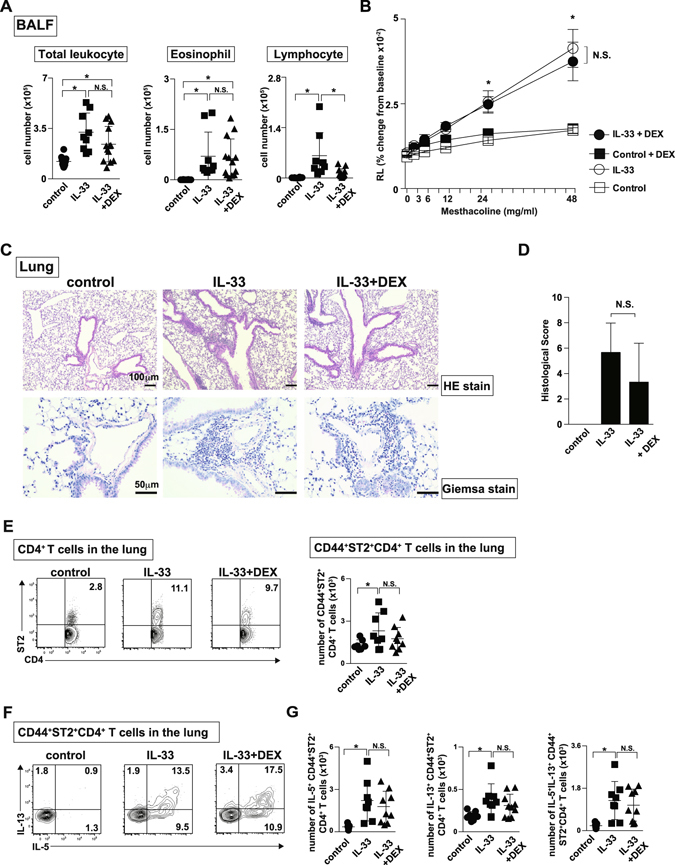
CD44^+^ST2^+^ CD4^+^ T cells showed steroid-resistance. IL-33 or saline (control) was administered intratracheally to BALB/c mice on Day 0 followed by intra-peritoneal administration of dexamethasone from Days 1 to 3. The indicated assays were performed on Day 10 or Day 14. (**A**) Cell numbers of total leukocytes (left), eosinophils (middle), and lymphocytes (right) in the BAL fluid are shown. The mean values ± SD are shown for three to five mice in each group. (**B**) Lung resistance (RL) was assessed in response to the increasing doses of methacholine. The mean values (four mice per group) are shown with SDs. *p < 0.05: one-way ANOVA. N.S. means not significant. (**C**) Representative histology of the lungs from mice as in **A**, fixed and stained with hematoxylin and eosin reagent (upper panels) or Giemsa reagent (lower panels). Scale bars: 100 μm in upper panel, 50 μm in lower panel. (**D**) Histological scores from Giemsa-stained samples are depicted. (**E**) Representative flow cytometry plots of ST2^+^CD4^+^ cells in the lung and spleen (left). Absolute numbers of CD44^+^ST2^+^CD4^+^ T cells in the lung from three groups are depicted. The mean values ± SD are shown for three to five mice in each group. (**F**) Representative intracellular staining profiles of IL-5 and IL-13 in CD44^+^ST2^+^CD4^+^ T cells. (**G**) Absolute numbers of indicated cytokine-producing CD44^+^ST2^+^CD4^+^ T cells in the lung from three groups are depicted. The mean values ± SD are shown for three to five mice in each group. Three independent experiments were performed, and similar results were obtained. *p < 0.05: one-way ANOVA.

Patients with eosinophilic pneumonia often suffer from recurrence of the diseases in association with the tapering of steroid therapy^8^. Airway inflammation induced by ILC2s or T helper (Th)17 cells has been appreciated as steroid-resistant pathological condition^20–22^. But the response of tissue-localized memory-type ST2^+^CD4^+^ T cells to steroid was not fully elucidated. Therefore, we wanted to evaluate the effect of steroid on the lung tissue-localized memory-type ST2^+^CD4^+^ T cells using our eosinophilic pneumonia model induced by intratracheal administration of IL-33. We examined the impact of dexamethasone (DEX) in the IL-33-induced eosinophilic lung inflammation model. Considering clinical situations for severe eosinophilic pneumonia, massive dose of DEX was administered after the induction of eosinophilic pneumonia (Fig. S5A). At the peak time point of eosionophilic inflammation (Day 10), DEX-treatment showed no impact on the augmented number of ST2^+^ IL-5- and IL-13-producing ILC2s, which suggested that ILC2s were steroid-resistant cell population as previously reported (Fig. S5B–D)^20^. Next, we investigated the effects of DEX in ST2^+^CD4^+^ T cells. The number of total inflammatory leukocytes and eosinophils in the BAL fluid remained roughly the same in the mice treated with IL-33 and DEX (Fig. 5A left and middle panels). In contrast, the number of lymphocytes in the BAL fluid was decreased in the mice treated with IL-33 and DEX (Fig. 5A right panel). DEX treatment showed small effects on enhanced airway hyperresponsiveness induced by IL-33 stimulation (Fig. 5B). Consistent with the profiles of the BAL fluid, the accumulation of eosinophils around the bronchioles and alveolar septa was detected in the lungs of mice treated with IL-33 and DEX (Fig. 5C and D). The numbers of CD44^+^ST2^+^ CD4^+^ T cells was almost equivalent in the lungs of mice treated with IL-33 and DEX compared to those in the mice treated with IL-33 alone (Fig. 5E). Furthermore, administration of DEX showed small impacts on the production of Th2 cytokines by CD44^+^ST2^+^ CD4^+^ T cells (Fig. 5F and G). These results indicate that the increased numbers of CD44^+^ST2^+^ CD4^+^ T cells and their IL-5- and IL-13-production were relatively resistant to treatment with DEX in our experimental setting.

## Discussion

Herein we highlighted the pathogenic role of memory-type ST2^+^CD4^+^ T cells in IL-33-induced eosinophilic airway inflammation. We found that intratracheal administration of IL-33 resulted in increased numbers of tissue-localized ST2^+^CD4^+^ T cells with enhanced production of IL-5 and IL-13. *Foxn1* ^*nu*^ mice showed severely attenuated IL-33-induced eosinophilic inflammation. In contrast, NSG mice with adoptive transfer of CD4^+^ T cells showed a similar level of eosinophilic inflammation to that in wild type mice. These results indicate that T cells rather than ILC2s are the major contributors shaping the pathology of IL-33-induced lung inflammation in our model. Furthermore, CD44^+^ST2^+^ CD4^+^ T cells appeared to be resistant to treatment with high-dose dexamethasone. Thus, our results highlighted the fact that memory-type ST2^+^CD4^+^ T cells are involved in the pathogenesis of steroid-resistant eosinophilic pneumonia induced by IL-33.

IL-33 is constitutively expressed on epithelial cells and other mesenchymal cells^23–26^. Chronic repeated exposure to various exogenous allergens or pathogens, such as tobacco smoke or inhaled irritant particles, prompts epithelial cells to release their stored IL-33 and IL-33 may directly regulate the local mucosa-residing immune cells, including tissue-resident memory T cells. We found increased numbers of lung tissue-localized memory-type ST2^+^CD4^+^ T cells accompanied with high production of IL-5 and IL-13 induced by the intratracheal administration of IL-33 (Fig. 1). Thus, it is most likely that lung tissue-localized memory-type ST2^+^CD4^+^ T cells respond to IL-33 directly and shape the pathology of allergic inflammation induced by IL-33.

ILCs are a recently established lymphocyte subset of tissue-resident immune cells that react promptly to various inner signals, such as cytokines, several pathogens, and certain allergens, without antigen-specific receptors^27, 28^. ILC2s preferentially reside in the lung with constitutive expression of ST2^27, 28^. In response to IL-33, ILC2s can be rapidly activated and produce large amounts of Th2 cytokines, such as IL-5 and IL-13^29, 30^. Therefore, ILC2s are recognized as the initial target cells for IL-33 in the lung^29, 31^. The pathogenic roles of ILC2s in the lung eosinophilic inflammation induced by IL-33 have been reported^32, 33^. In our experimental setting, both ILC2s and memory-type ST2^+^CD4^+^ T cells responded to IL-33 in the lung tissue. Interestingly, we observed a distinct pattern in the time course of activation of ILC2s and memory-type ST2^+^CD4^+^ T cells after IL-33 stimulation *in vivo*. ILC2s promptly and vigorously reacted, and increased numbers of IL-5 and IL-13-producers were detected on Day 3 after IL-33 stimulation (Fig. S1K and L). In contrast, memory-type ST2^+^CD4^+^ T cells took 10 days to increase numbers of IL-5- and IL-13-producing cells (Fig. 1H and I). Moreover, in spite of sufficient reaction of ILC2s, T cell-deficient *Foxn1* ^*nu*^ mice showed poor eosinophilic inflammation at Day 14, when the tissue injury attained to the peak in wild type mice after intratracheal administration of IL-33. Based on the time course of cytokine-producing in ILC2s, ILC2s may have a role in the eosinophilic lung inflammation at the early time point in T cell-deficient *Foxn1* ^*nu*^ mice. In fact, ILC2s appear not to be involved in the pathogenicity of IL-33-induced eosinophilic inflammation at the late time point in our experiment (Fig. 4). Taken together, the current study indicated the requirement of T cells in the pathology of eosinophilic inflammation in the lung (Fig. 3), and memory-type ST2^+^CD4^+^ T cells are the major contributors shaping the pathology of IL-33-induced eosinophilic lung inflammation particularly at the late time point.

Corticosteroids are a key medicine used to treat various types of inflammatory diseases, including allergy and autoimmune diseases^34^. However, a substantial number of patients are refractory to an adequate dose of corticosteroids and suffer from uncontrolled symptoms, although the underlying mechanisms are still unclear^34^. Patients with certain types of eosinophilic pneumonia are also poorly responsive to corticosteroids therapy^8, 35^. In particular, among patients with chronic-type eosinophilic pneumonia, eosinophilia often recurs as the corticosteroid dose is tapered. In an experimental model of bronchial asthma, airway inflammation induced by ILC2s or T helper (Th)17 cells has been reported to be steroid-resistant^20–22^. In the present study, we showed that treatment with high-dose DEX showed little effect on the numbers of memory-type ST2^+^CD4^+^ T cells and their production of Th2 cytokines (Fig. 5E–G). In our IL-33-induced eosinophilic lung inflammation model, eosinophil infiltration was observed both in the peribronchiolar area and in the alveolar spaces, and the vascular permeability was enhanced (Fig. 2). These characteristic inflammatory changes are reminiscent of those in the lung of patients with eosinophilic pneumonia. Thus, memory-type ST2^+^CD4^+^ T cells are steroid-resistant and appear to play a critical role in steroid-resistant types of eosinophilic lung inflammation, such as chronic-type eosinophilic pneumonia. Interestingly, substantial numbers of previous studies have identified *ST2*, which encodes ST2, as s susceptible gene in asthma and chronic rhinosinusitis (CRS)^7, 36–39^. CRS is an upper airway inflammatory disease that develops as a consequence of chronic inflammation with distinct cytokine patterns^40^. CRS with polyps is classified as ECRS and non-eosinophilic CRS (NECRS) based on the diagnostic histological changes^41^. Patients with ECRS are often associated with asthma, and the polyps of these patients show massive infiltration of eosinophils, suggesting that type 2 immunity is deeply involved in the pathogenesis of ECRS^41^. Furthermore, the Th2 bias appears to be associated with comorbid asthma and recurrence after canonical treatment^42^. We reported the increased expression of *ST2* in memory-type CD4^+^ T cells from polyps of patients with ECRS compared to those in memory-type CD4^+^ T cells from polyps of patients with NECRS^5^. Thus, ST2^+^CD4^+^ T cells locally exist in patients with ECRS and appear to be involved in the pathogenicity of ECRS. Further studies are needed to clarify the systemic involvement of ST2^+^CD4^+^ T cells in ECRS patients. We await the findings of studies addressing the direct relationship between ST2^+^CD4^+^ T cells and the efficacy of corticosteroid therapy in ECRS patients.

In conclusion, memory-type ST2^+^CD4^+^ T cells play crucial roles in the pathology of eosinophilic inflammation in the lung. Furthermore, memory-type ST2^+^CD4^+^ T cells appeared to be resistant to steroid treatment *in vivo*. Thus, the process of IL-33-induced activation of memory-type ST2^+^CD4^+^ T cells may be a potential therapeutic target for steroid-resistant recurrent eosinophilic lung inflammation, including eosinophilic pneumonia.

## Methods

### Ethical Approval

The research proposals were reviewed by the ethics committee for animals at Jichi Medical University (registration number: 15204) and Chiba University (registration number: 28–182). All animal experiments were performed in accordance with the Declaration of Helsinki conventions for the use and care of animals and guidelines of both universities. All animal procedures and experimental protocols were carried out in accordance with the approved guidelines and institutional regulations.

### Mice

BALB/c and *Foxn1* ^*nu*^ mice were purchased from CLEA Japan (Tokyo, Japan), *Kit* ^*W*^ */Kit* ^*W-v*^ mice, *Gata1* ^*tm6Sho*^ mice and NSG mice were purchased from Japan SLC (Shizuoka, Japan), and Jackson Laboratory (Bar Harbor, ME, USA), respectively. All mice were used at 6–10 weeks old and were maintained under specific-pathogen-free conditions.

### Mouse model of eosinophilic lung inflammation with enhanced vascular permeability

Recombinant mouse IL-33 (5 μg; R&D Systems, Minneapolis, MN, USA) dissolved in 50 μl sterile saline was intratracheally administered on Day 0. BAL fluid were obtained and analyzed at the indicated time points. The concentration of albumin was measured by a N-assay TIA Micro Alb (Nittobo, Tokyo, Japan).

### Lung mononuclear cell preparation

Lung mononuclear cells were obtained as previously described ^2^. Cells were stimulated for 6 hours with Phorbol-12-myristate 13-acetate (PMA) (50 ng/ml), ionomycin (0.5 μM), and monensin (2 μM), and pre-incubated with anti-CD16/32 antibody (clone 2.4G2; TONBO Bioscience, Kobe, Japan). For intracellular staining, cells were fixed with Transcription Factor Buffer Set (BD Bioscience, San Jose, CA, USA) in accordance with the manufacturer’s protocol.

### Intravenous staining for CD4^+^ T cells localized in the vasculature in the lung

For intravenous staining of CD4^+^ T cells, 0.5 μg FITC-conjugated anti-CD4 antibody (RM4-4) diluted in 50 μl PBS was administered via the tail vein. At three minutes after the injection, mice were anesthetized with isoflurane and sacrificed.

### Adoptive transfer of CD4 T cells

CD4^+^ T cells from spleens of 6- to 8-week-old BALB/c mice or Thy1.1 transgenic mice were purified by negative selection and magnetic separation (Miltenyi Biotec). CD4^+^ T cells (1.2 × 10^7^ cells) were transferred i.v. into NSG mice that were subsequently intranasal administered IL-33 as indicated in Supplementary Figure 4A.

### Flow cytometry and antibodies

Cell surface staining were performed with the following antibodies: anti-CD3ε, and anti-CD4, anti-CD11b, anti-CD11c, anti-CD19, anti-B220, and anti-Ly-6G (TONBO Bioscience, San Diego, CA, USA); anti-IL-33R (ST2), anti-TCRγδ, anti-CD90.2, and anti-CD45 (BioLegend, San Diego, CA, USA); anti-TCRβ, anti-CD25, anti-CD44, anti-CD62L, anti-CD69, anti-CD103, anti-NK1.1, and anti-TER119 (BD Bioscience); and anti-CD127 (eBioscience, San Diego, CA, USA). The same procedures were conducted with the following antibodies for the intracellular staining: anti-IL-4 and anti-IL-5 (BD Bioscience); and anti-IL-13 (eBioscience). Fixable Viability Dye (eBioscience) was used to exclude dead cells. All samples were analyzed using the FACS Verse (BD Bioscience) and Flow Jo software programs (Tree Star Inc., San Carlos, CA, USA).

### Histology and immunohistochemistry

Pathological changes were evaluated by Haematoxylin & Eosin (HE), Giemsa staining, and immunofluororescent staining as previously described^2, 43^. Anti-mouse T1/ST2 monoclonal antibody (Thermo Fisher, Waltham, MA. USA), anti-mouse CD4 (BioLegend), anti-mouse CD44 (BioLegend), and 4′,6-diamidino-2-phenylindole (DAPI) (Thermo Fisher) were used for immunofluororescent staining.

### Histological score

Histological scores were determined based on the presence of the following^44^: perivascular eosinophils (0–2), alveolar eosinophils (0–2), perivascular mononuclear cells (0–2), alveolar mononuclear cells (0–2), epithelial desquamation (normal = 0; elongation/distortion = 1; elongation, infolding, and narrowing = 2; loss of cells with broken airways = 3). Pooled data are shown from two independent experiments with four mice per group. The mean values are shown with standard deviations (SDs). *p < 0.05: one-way ANOVA. N.S. means not significant.

### Statistical analysis

We expressed the data as the mean ± SD. Mann-Whitney U test, and a one-way analysis of variance (ANOVA) with Tukey’s test were performed for statistical analysis with the GraphPad Prism version 5.0 (Graph-Pad Software, Inc. San Diego, CA, USA). A *p*-value < 0.05 was considered statistically significant.

## Electronic supplementary material

Supplementary Information
