# Supplementary material for: Memory-type ST2+CD4+ T cells participate in the steroid-resistant pathology of eosinophilic pneumonia
Source: Sci Rep. 2017 Jul 28;7:6805. doi: 10.1038/s41598-017-06962-x (PMC5533714; doi:10.1038/s41598-017-06962-x)

Supplementary Information

Memory-type ST2$^+$CD4$^+$ T cells participate in the steroid-resistant pathology of eosinophilic pneumonia

Naoko Mato [1,2], Kiyoshi Hirahara [2], Tomomi Ichikawa [2], Jin Kumagai [2], Masayuki Nakayama [1], Hideaki Yamasawa [1], Masashi Bando [1], Koichi Hagiwara [1], Yukihiko Sugiyama [1,3], and Toshinori Nakayama [2,4*]

1 Division of Pulmonary Medicine, Department of Internal Medicine, Jichi Medical University, 3311-1 Yakushiji, Shimotsuke-city, Tochigi 329-0434, Japan

2 Department of Immunology, Graduate School of Medicine, Chiba University, 1-8-1 Inohana, Chuo-ku, Chiba 260-8670, Japan

3 Department of Respiratory Medicine, Nerima-Hikarigaoka Hospital

4 AMED-CREST, AMED, 1-8-1 Inohana Chuo-ku, Chiba 260-8670, Japan

* Corresponding author: Toshinori Nakayama

Telephone: +81-43-226-2200 Fax: +81-43-227-1498

Mailing Address: 1-8-1 Inohana, Chuo-ku, Chiba-shi, Chiba, 260-8670 Japan

E-mail: tnakayama@faculty.chiba-u.jp

Supplemental Figure 1

(A) Representative flow cytometry plots of $ST2^+CD4^+$ T cells in the lung and spleen. (B) The percentage of $ST2^+CD4^+$ T cells among total $CD4^+$ T cells in the lung was depicted. (C) Representative flow cytometry plots of $ST2^+$ and $ST2^-$ $CD4^+$ T cells in the lung. (D) The expression of CD44 (left) and CD62L (right) on $ST2^+$ (red) and $ST2^-$ (blue) $CD4^+$ T cells in the lung are shown. The gray-shaded histogram represents the corresponding isotype-matched control staining. Two independent experiments were performed with similar results. (E) Representative flow cytometry plots of $CD4^+$ T cells in the spleen and peripheral blood after the intravenous injection of anti-CD4 antibody. The isotype control represents the corresponding isotype-matched control antibody injection. Control: saline-treated mice, IL-33: IL-33-treated mice. (F) The protocol of IL-33-treatment and the following analyses are depicted. IL-33 (5 µg) was administered intratracheally. Analyses by flow cytometry were performed at the indicated time points. (G) Representative flow cytometry plots of intracellular staining profiles of IL-4 and IL-5 in $CD44^+ST2^+CD4^+$ T cells after saline(control) or IL-33-administration (left panel). Absolute numbers of IL-4-producing $CD44^+ST2^+CD4^+$ T cells in the lung from the two groups are depicted (right panel). The mean values ± SD are shown for four mice in each group. (H) Lung ILC2s were identified as follows: live cells were separated by viability dye, and lineage marker-negative and $CD45^+$ and

Thy1.2$^+$ cells were selected and ultimately confirmed by positive staining by CD25 and CD127. (I) Representative flow cytometry plots of ST2$^+$ILC2s in the lung at the indicated time points are depicted after IL-33-administration. (J) Absolute cell numbers of ST2$^+$ ILC2s in the lung from the two groups at the indicated time points are depicted. (K) Representative intracellular staining profiles of IL-5 and IL-13 of ST2$^+$ILC2s. (L) Absolute cell numbers of indicated cytokine-producing ST2$^+$ILC2s are depicted. The mean values ± SD are shown for three to five mice in each group. Two independent experiments were performed with similar results (C-E, G-L). Three independent experiments were performed with similar results (A, B). $^*p < 0.05$; Mann-Whitney U test.

Supplemental Figure 2

(A) Experimental protocol of intratracheal IL-33-treatment and subsequent analyses. IL-33 (5 μg) was administered intratracheally, and the BAL and peripheral blood samples were analyzed at the indicated time points. (B) The numbers of total leukocytes (left), eosinophils (middle), and lymphocytes (right) at the indicated time points in peripheral blood are shown. The mean values ± SD are shown for four to seven mice in each group. Pooled data from two independent experiments were provided in (B). One-way ANOVA was performed.

Supplemental Figure 3

IL-33 was administered to *Gata1*^{tm6Sho} mice (deficient in eosinophils), *Kit*^{W}/*Kit*$^{W-v}$ mice (deficient in mast cells and basophils). The BAL fluid was analyzed, and a histological examination was performed. (A) Numbers of total leukocytes and eosinophils in the BAL fluid in *Gata1*^{tm6Sho} mice and *Kit*^{W}/*Kit*$^{W-v}$ mice are depicted. (B) Representative microscopic pictures of the lungs stained with hematoxylin-eosin from *Gata1*^{tm6Sho} mice are shown. (C) Histological scores from HE stained samples are depicted. (D) Representative microscopic pictures of the lungs stained with hematoxylin-eosin from *Kit*^{W}/*Kit*$^{W-v}$ mice are shown. (E) Histological scores from HE stained samples are depicted. (F) Representative flow cytometry plots describing the process of preparation of ST2^{+} ILC2 cells in the lung from WT mice and *Foxn1*^{nu} mice after saline (control) or IL-33-administration are shown. (G) Absolute cell numbers of ST2^{+}ILC2 cells in the lung from WT mice and *Foxn1*^{nu} mice are depicted. (H) Representative intracellular staining profiles of IL-5 and IL-13 in ST2^{+}ILC2 cells from WT mice and *Foxn1*^{nu} mice (upper panel). Absolute cell numbers of the indicated cytokine-producing ST2^{+}ILC2 cells are depicted from WT mice and *Foxn1*^{nu} mice (lower panel). The mean values ± SD are shown for each group (A, G, and H). Representative data were obtained from two individual experiments (A), and pooled data (n=5-7) from two independent experiments were provided in (G, and H). WT: wild type BALB/c mice, *Kit*$^{+}$/*Kit*$^{+}$:

littermate of *Kit*^{W}/*Kit*$^{W-v}$ mice. *$p<0.05$: One-way ANOVA.

Supplemental Figure 4

(**A**) Experimental protocol of adoptive transfer prior to the IL-33-administration and subsequent analyses is depicted. Naïve CD4^{+} T cells obtained from spleen of Thy1.1 congenic mice or BALB/c mice were transferred to NSG mice, and saline (control) or IL-33 (5 μg) was administered intratracheally on the next day, and the FACS was performed at day 10, and BAL, AHR and histological analyses were performed at day14. (**B**) To distinguish lung tissue-localized CD4^{+} T cells and blood-borne CD4^{+} T cells, anti-CD4 antibody was injected intravenously and analyzed three minutes later. In the CD4^{+} T cells in the lung, injected antibody-unstained cells (CD4 (i.v.)$^{-}$ cells) indicate lung tissue-localized CD4^{+} T cells. Representative flow cytometry plots of ST2^{+}CD4 (i.v.)$^{-}$ CD4^{+} T cells in the lung of wild type mice (BALB/c) and NSG mice with adoptive transfer at day10. (**C**) Percentage of ST2^{+}CD4^{+} T cells in the lung from three groups in Figure **4A** are depicted. (**D**) Representative flow cytometry plots of lung tissue-localized CD4^{+} T cells in the lung at day10 are depicted. The histogram represents the expression of CD44 on lung tissue-localized CD4^{+} T cells. *$p<0.05$: one-way ANOVA.

Supplemental Figure 5

(A) The protocol of administration of IL-33 with or without dexamethasone is depicted. (B) Absolute cell numbers of the $ST2^{+}ILC2$ cells are depicted from three groups. The mean values ± SD are shown for eight to ten mice in each group. (C) Representative intracellular staining profiles of IL-5 and IL-13 in $ST2^{+}ILC2$ cells. (D) Absolute numbers of indicated cytokine-producing $ST2^{+}ILC2$ cells in the lung from three groups are depicted. The mean values ± SD are shown for eight to ten mice in each group. Representative data were obtained from two individual experiments (C), and pooled data (n=8-10) from three independent experiments were provided in (B, D). $^{*}p<0.05$: one-way ANOVA.

Supplemental Figure 1

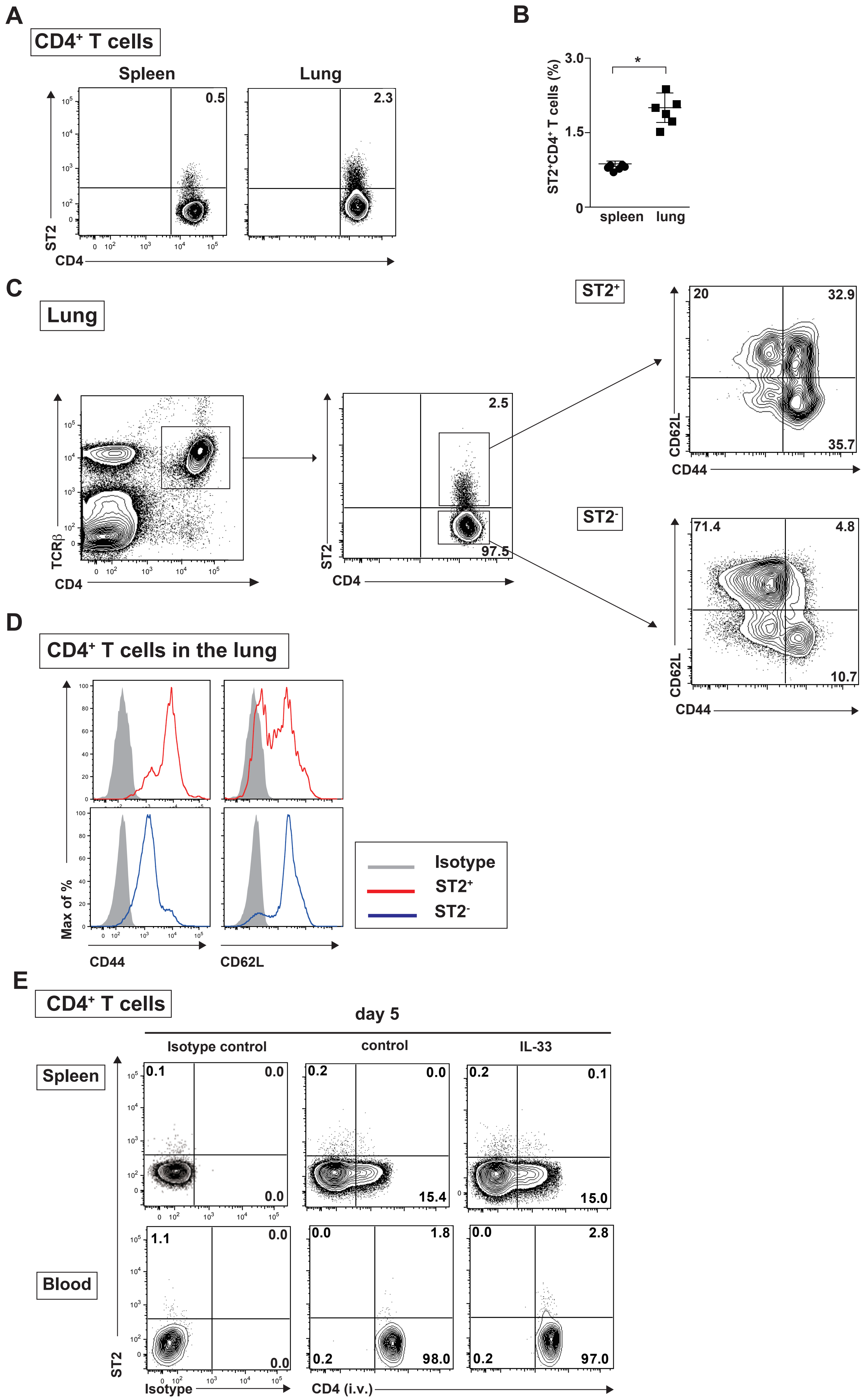

Supplemental Figure 1 (Continued)

F

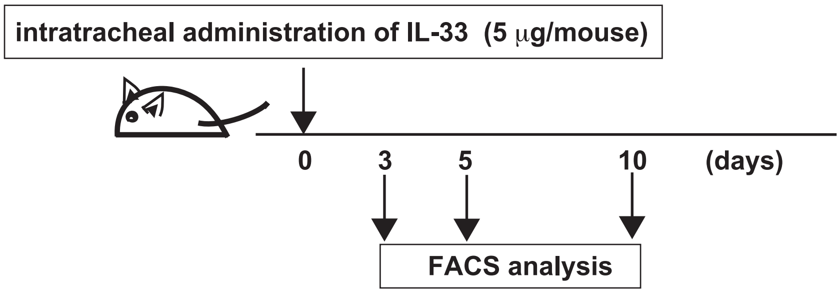

G

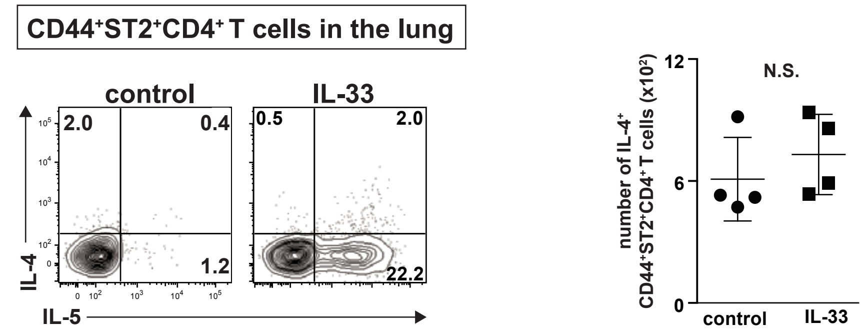

H

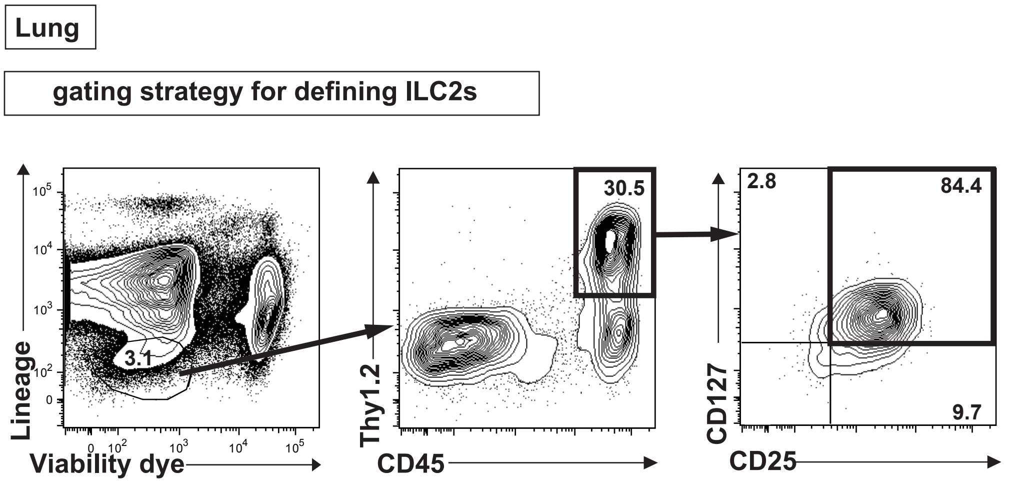

I

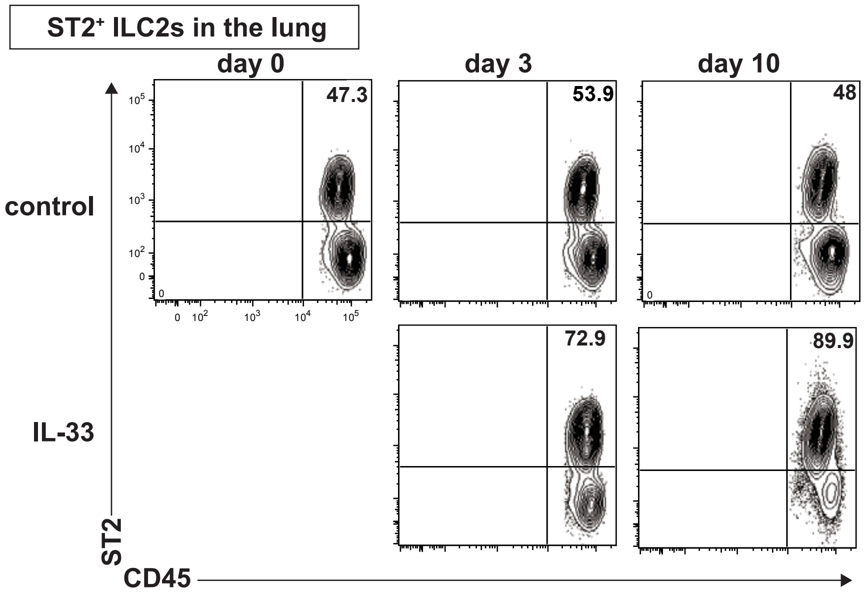

J

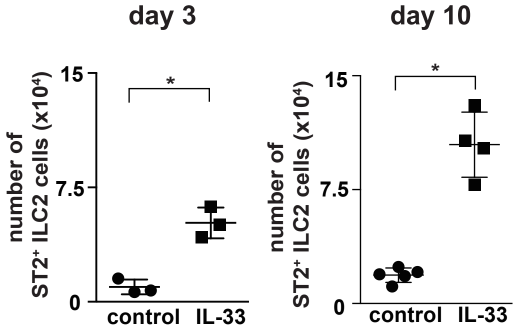

Supplemental Figure 1 (Continued)

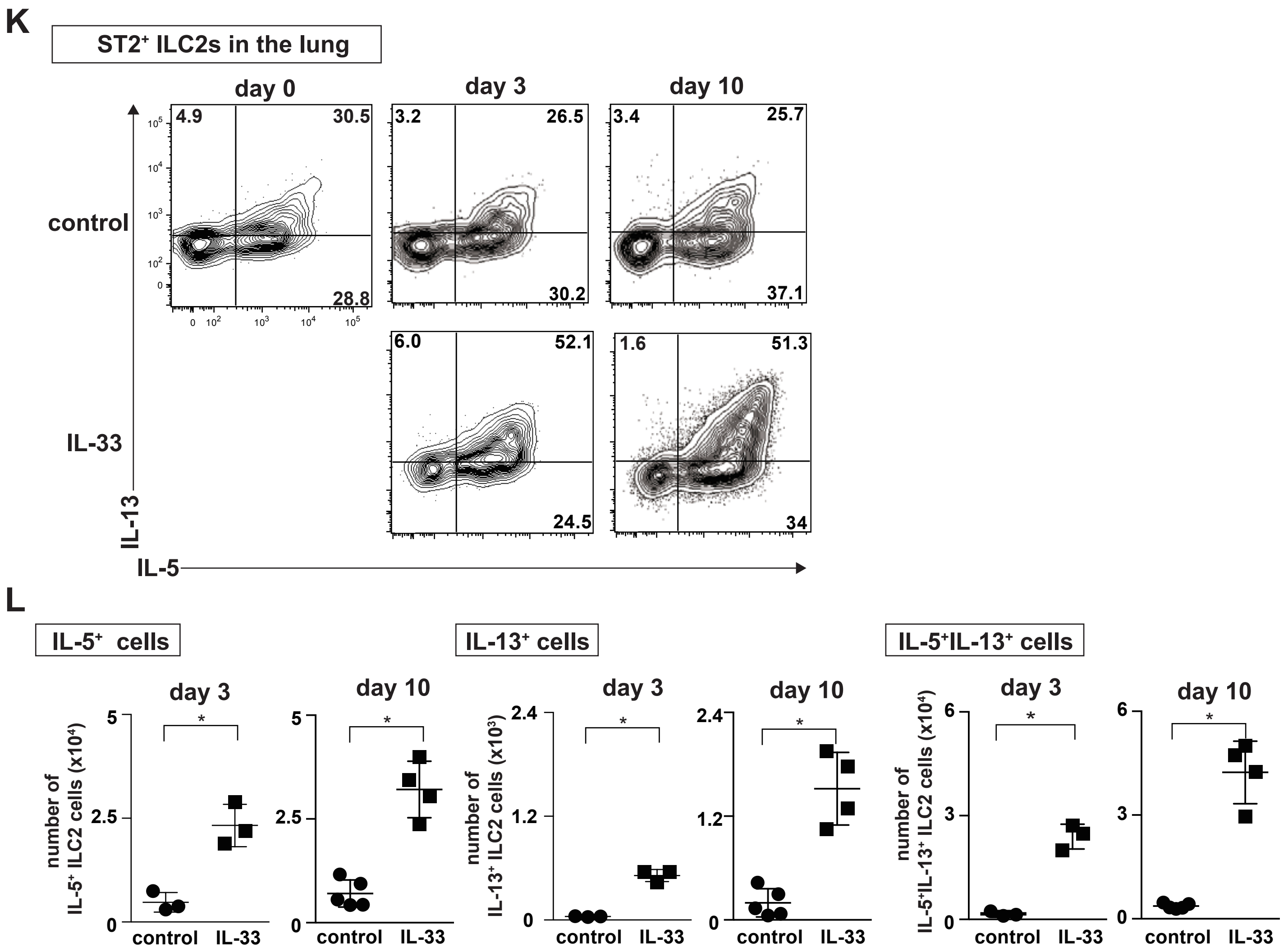

Supplemental Figure 2

A

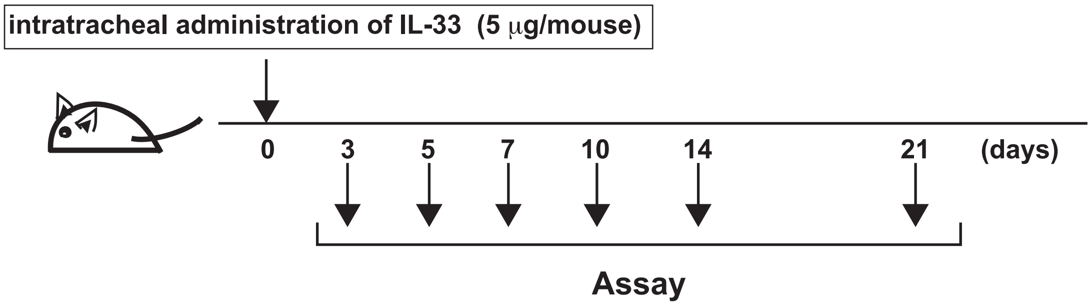

B

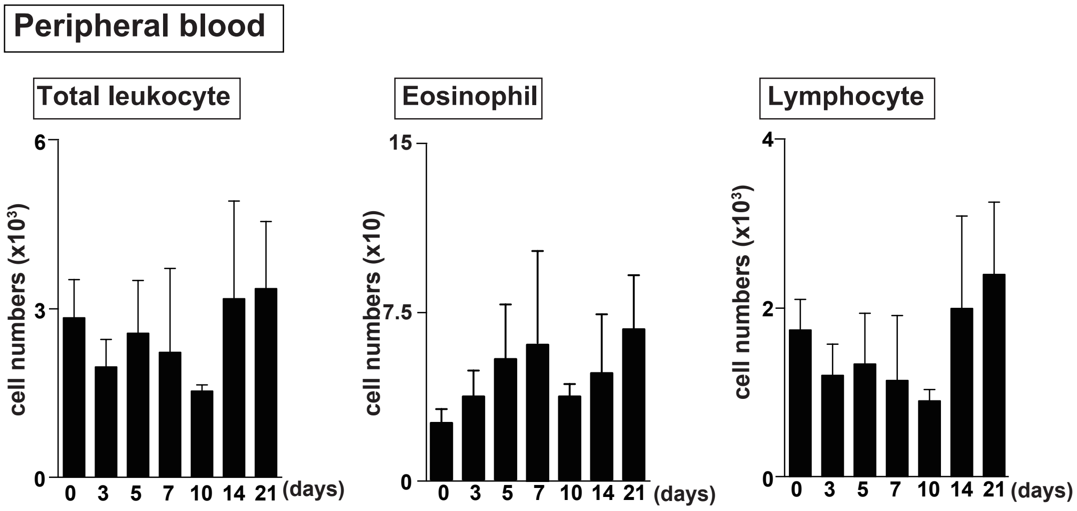

Supplemental Figure 3

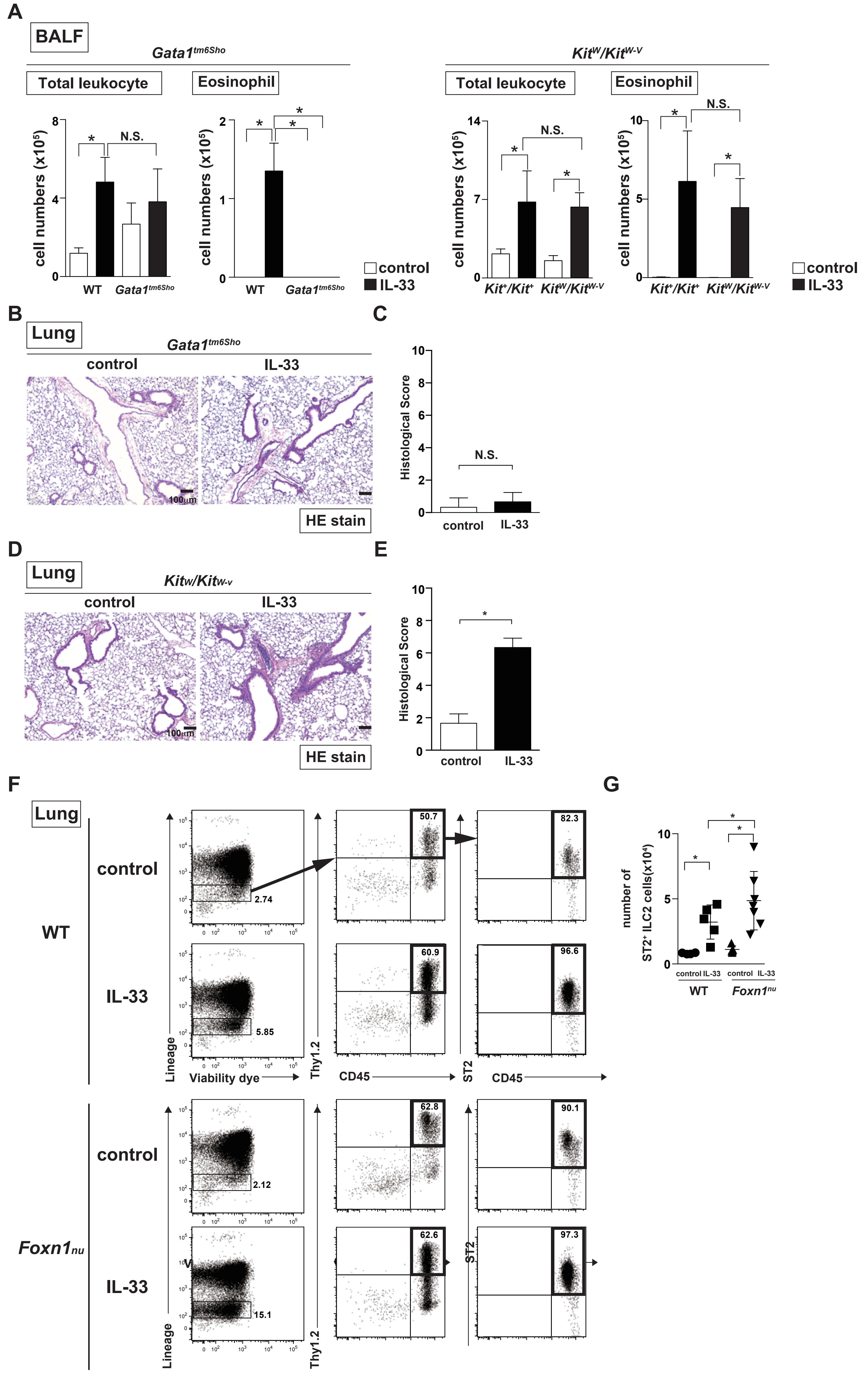

Supplemental Figure 3 (Continued)

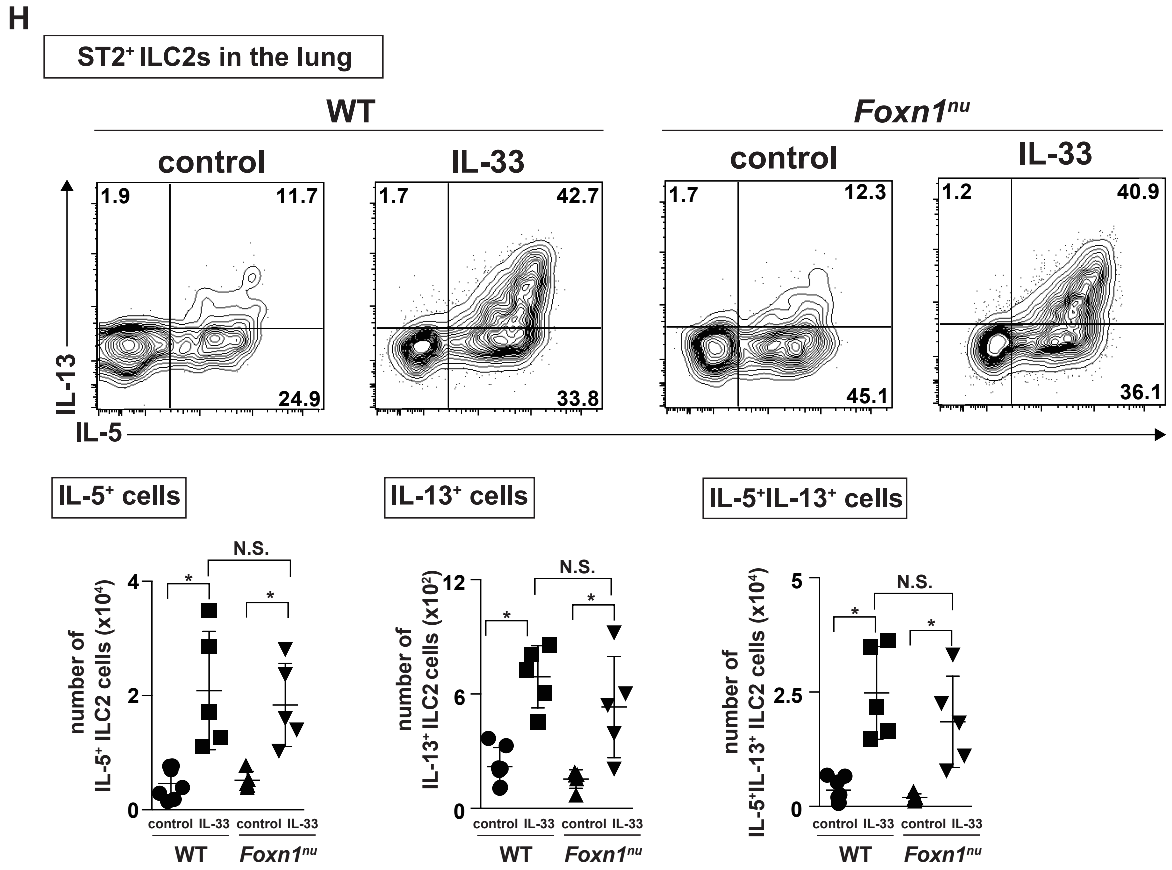

Supplementary Figure 4

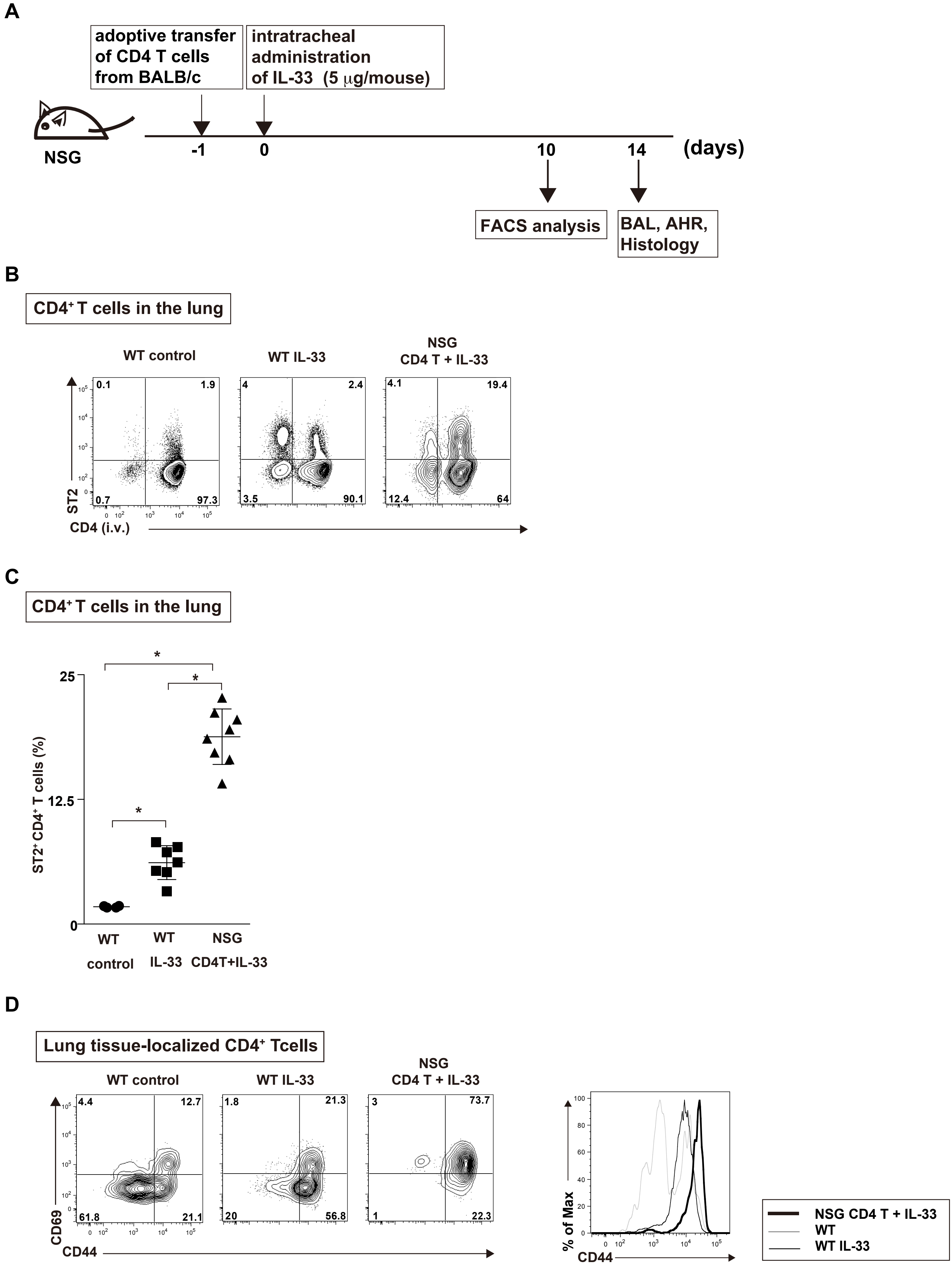

Supplemental Figure 5

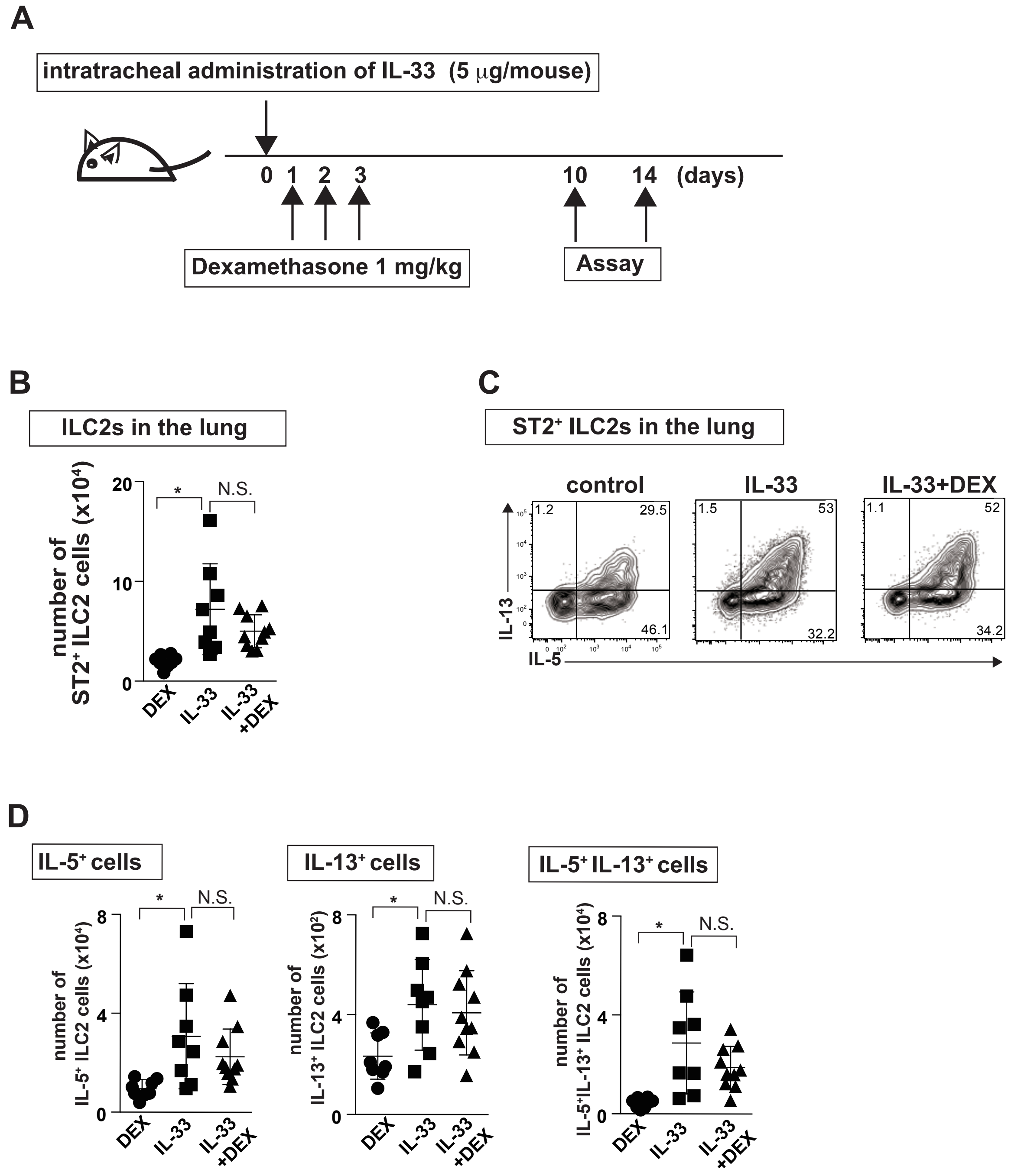

Supplement: Supplementary file 1 — Supplementary Information [file 41598_2017_6962_MOESM1_ESM.pdf]
